# Serum CEACAM6 and CEACAM8 as predictors of poor outcome in pediatric *Mycoplasma pneumoniae* pneumonia: A single-center prospective cohort study

**DOI:** 10.1097/MD.0000000000048137

**Published:** 2026-04-10

**Authors:** Shenghua Ge, Lingyan Ma, Dongqing Song

**Affiliations:** a Department of Pediatrics, Shijiazhuang People’s Hospital, Shijiazhuang, Hebei Province, China.

**Keywords:** biomarkers, CEACAM6, CEACAM8, *Mycoplasma pneumoniae* pneumonia, pediatric, prognosis

## Abstract

This study aimed to evaluate the prognostic utility of serum carcinoembryonic antigen-related cell adhesion molecule 6 (CEACAM6) and CEACAM8 levels in children with *Mycoplasma pneumoniae* pneumonia (MPP). This single-center prospective cohort study enrolled 364 children with confirmed MPP and 160 age-matched healthy controls between February 2023 and May 2025. Serum levels of CEACAM6, CEACAM8, lactate dehydrogenase (LDH), D-dimer, interleukin-6, C-reactive protein, and myeloperoxidase were measured at admission using enzyme-linked immunosorbent assay. Patients were classified into good- (n = 295) or poor-prognosis (n = 69) groups after 1 week of standardized azithromycin therapy based on clinical, radiological, and spirometric criteria. Multivariable logistic regression was used to identify independent predictors of poor outcome. Receiver-operating characteristic curve analysis assessed the predictive performance of CEACAM6 and CEACAM8, individually and in combination, with corresponding areas under the curve. Correlations with inflammatory markers were evaluated using the Spearman rank correlation test. Children with poor outcomes had significantly higher CEACAM6 and CEACAM8 levels at admission (both *P* < .001). Multivariable analysis identified elevated CEACAM6 (odds ratio = 1.12, 95% confidence interval = 1.05–1.19) and CEACAM8 (odds ratio = 1.09, 95% confidence interval = 1.03–1.15) as independent predictors of poor prognosis. Receiver-operating characteristic analysis demonstrated areas under the curve of 0.771 for CEACAM6, 0.755 for CEACAM8, and 0.826 for their combination. Both biomarkers correlated positively with interleukin-6, C-reactive protein, and myeloperoxidase (all *P* < .01) and declined significantly after treatment. Serum CEACAM6 and CEACAM8 are independently associated with poor outcomes in pediatric MPP. Their combined use enhances prognostic accuracy and may support early risk stratification in clinical practice.

## 1. Introduction

*Mycoplasma pneumoniae* (MP) is a leading pathogen of community-acquired pneumonia in the pediatric population. The disease predominantly affects school-aged children and adolescents, who typically present with fever, cough, and other respiratory symptoms; consequently, *Mycoplasma pneumoniae* pneumonia (MPP) has emerged as an important global public health challenge.^[1]^ Early clinical experience indicated that standard macrolide therapy achieves clinical cure within approximately 1 week in the majority of cases. However, the escalating prevalence of macrolide resistance is undermining therapeutic efficacy, exacerbating disease severity through increased risk of serious complications, and driving a more aggressive, potentially fatal clinical course.^[2]^ Despite advances in comprehensive management strategies, a subset of children experience progressive pulmonary pathology and, influenced by multiple determinants, exhibit poor long-term outcomes.^[3]^ Therefore, identification of novel biomarkers capable of predicting disease trajectory is essential to guide individualized therapeutic decisions. Studies have demonstrated that plasma levels of soluble B7-H3 and chitinase-3-like protein 1 exhibit high early predictive value for refractory *Mycoplasma pneumoniae* pneumonia (RMPP) and may serve as potential biomarkers for identifying RMPP.^[4]^ Conversely, traditional inflammatory markers such as interleukin-6 (IL-6) serve as potential biomarkers for assessing MPP severity and prognosis; however, they are susceptible to influences from glucocorticoid therapy and other factors, resulting in decreased levels.^[5]^ In addition to these, neutrophil-to-lymphocyte ratio, platelet-to-lymphocyte ratio, lactate dehydrogenase (LDH), D-dimer (D-D), and C-reactive protein (CRP) have been demonstrated to be closely associated with MPP severity and prognosis. While current MPP biomarker research covers cellular, protein, cytokine, and nucleic acid dimensions, reliable biomarkers for early prediction of adverse outcomes are still lacking. Thus, identifying novel biological markers is crucial for improving prognosis and clinical management in pediatric MPP.^[6]^

The carcinoembryonic antigen-related cell adhesion molecule (CEACAM) family plays diverse immunomodulatory roles in both inflammatory and infectious disease contexts.^[7]^ Among its members, CEACAM6 and CEACAM8 have emerged as pivotal players in diverse pathological processes.^[8]^ CEACAM6 is constitutively expressed on epithelial and myeloid cells, where it modulates cell adhesion, signal transduction, and innate immune responses.^[9]^ Upregulation of CEACAM6 attenuates heme oxygenase-1–mediated antioxidant defenses, thereby potentiating tissue injury in chronic obstructive pulmonary disease.^[10]^ CEACAM8 is selectively expressed by neutrophils and eosinophils and governs granulocyte adhesion and activation.^[11]^ Transcriptomic profiling of COVID-19 identified CEACAM8 among the most significantly upregulated genes, with circulating levels distinguishing infected individuals from healthy controls and implicating CEACAM8-driven neutrophil dysfunction in disease severity.^[12]^

At present, the prognostic significance of serum CEACAM6 and CEACAM8 in pediatric MPP remains undefined. This study was designed to delineate the association between these glycoproteins and adverse clinical outcomes, and to appraise their utility as predictive biomarkers that could inform early intervention and personalized therapeutic strategies.

## 2. Materials and methods

### 2.1. Study participants

A prior single-center study reported a 16.10% incidence of unfavorable outcomes in children with MPP who received azithromycin.^[13]^ Treating this figure as the expected proportion, we set the absolute precision (δ) at 0.05 (95% confidence interval [CI] width = 10%) and α = 0.10 (two-tailed). Power analysis using the “Confidence Interval for One Proportion” module of PASS 15.0 (NCSS Statistical Software, Kaysville) indicated that a minimum of 226 MPP cases were required to achieve the desired precision.

**Figure 1.**
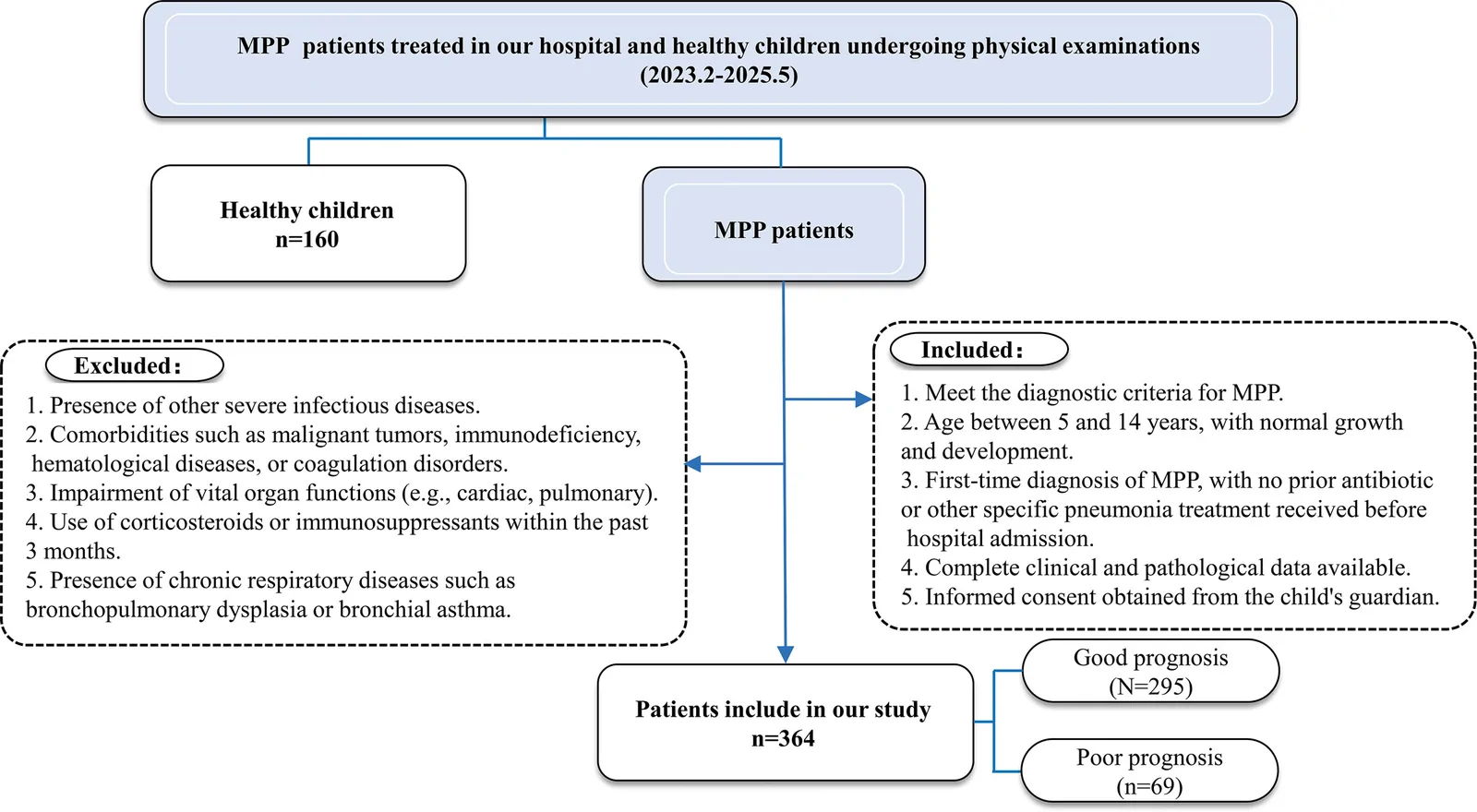
Participant flow diagram. MPP = *Mycoplasma pneumoniae* pneumonia.

Between February 2023 and May 2025, 364 children admitted to our hospital with a definitive diagnosis of MPP were prospectively enrolled as the observation cohort; 160 age-matched healthy children undergoing routine health checks served as controls. The participant flow diagram is presented in Figure 1. The study was conducted in accordance with the Declaration of Helsinki and was approved by the Ethics Committee of Shijiazhuang People’s Hospital (Approval No. 2023032). Written informed consent was obtained from parents or legal guardians of all participants.

### 2.2. Diagnostic, inclusion, and exclusion criteria

Diagnostic criteria: MPP was defined according to the 2023 Chinese guidelines for the diagnosis and treatment of childhood MPP^[14]^: acute respiratory illness (fever, cough, wheeze, or tachypnea), pulmonary infiltrates on chest imaging, positive serum MP-immunoglobulin M (particle agglutination titer ≥ 1:160) OR detection of MP-DNA/RNA in nasopharyngeal swab OR ≥4-fold rise in paired serum MP antibody titers.

Inclusion criteria: fulfillment of the above MPP definition, age 5 to 14 years with normal growth and development, first episode of MPP without prior antibiotic or anti-pneumonia therapy before admission, complete clinical and laboratory data available, and written informed consent from parent/legal guardian.

Exclusion criteria: coexisting severe infectious disease; malignancy, primary immunodeficiency, hematologic disorder, or coagulopathy; significant cardiac, pulmonary, or other vital organ dysfunction; systemic glucocorticoid or immunosuppressive use within 3 months; and chronic lung disease such as bronchopulmonary dysplasia or bronchial asthma.

### 2.3. Treatment protocol

Supportive management, encompassing oxygen, nebulized bronchodilators, antipyretics, and mucolytics, was provided to all participants. Azithromycin (Jilin Jinsheng Pharmaceutical Co., Ltd., Meihekou, Jilin Province, China; NMPA H20223970) therapy was instituted as a 7-day course: 500 mg intravenously once daily for 2 days, followed by 500 mg orally once daily for 5 days.

### 2.4. Clinical data

Baseline demographics (age, sex, body weight), white-cell count, neutrophil count, and serum LDH were recorded. Intergroup comparisons included age, sex, perinatal history, duration and peak of fever, Clinical Pulmonary Infection Score (CPIS),^[15]^ and length of hospital stay.

### 2.5. Laboratory assays

Within 3 hours of admission, 5 mL of fasting venous blood was collected, centrifuged at 3000 × g for 15 minutes, and the serum was aliquoted. Samples were either assayed immediately after 1:2 dilution (50 μL serum + 50 μL diluent) or stored at −20°C without repeated freeze–thaw cycles. All procedures followed the manufacturers’ protocols.

Serum levels of LDH, D-D, CEACAM6, CEACAM8, IL-6, CRP, and myeloperoxidase (MPO) were quantified by sandwich enzyme-linked immunosorbent assay (ELISA) using kits from Shanghai Enzyme-linked Biotechnology Co., Ltd. (Shanghai, China; catalogue nos. ml106625, ml057585, ml105729, ml105730, ml058097, ml027874, and ml106104, respectively). Anti-MP antibody titers were determined by passive agglutination. Complete blood counts were obtained on a Sysmex XN-1000 (Sysmex, Kobe, Japan) automated hematology analyzer.

Blinding: Specimens were identified only by a unique random code; laboratory personnel performing ELISA and data verification were unaware of clinical diagnoses and group allocation. Because of the observational design, blinding of participants or attending physicians was not feasible.

### 2.6. Outcome assessment

One week after the initiation of therapy, a single senior pediatrician (blinded to laboratory results) classified each patient according to early treatment response criteria.^[16]^

Good-prognosis group (n = 295): complete resolution of fever, cough, and wheeze; ≥50% reduction in pulmonary infiltrates on chest imaging (mean of 2 independent, blinded radiologists); and clinical stability without need for therapeutic escalation.

Poor-prognosis group (n = 69): persistence or worsening of clinical signs (persistent fever ≥ 38°C for ≥7 days or deteriorating respiratory distress); computed tomography evidence of disease progression (expansion of consolidation, new-onset atelectasis, pleural effusion, or necrotizing pulmonary lesions); and/or requirement for treatment escalation.

All data were entered independently by 2 investigators; discrepancies >5% were resolved by a third reviewer.

### 2.7. Statistical analysis

Statistical analyses were performed using IBM SPSS Statistics for Windows, version 26.0 (IBM Corp., Armonk). Categorical variables were expressed as n (%) and compared by the χ^2^ test. Continuous data were presented as mean ± SD or median (interquartile range) and analyzed using the *t* test or Mann–Whitney *U* test, as appropriate. Independent predictors were identified by multivariable logistic regression. The prognostic performance of CEACAM6 and CEACAM8 was evaluated by receiver-operatingcharacteristic (ROC) analysis, and their correlations with inflammatory markers were assessed using Spearman correlation. A two-tailed *P* < .05 was considered statistically significant.

## 3. Result

### 3.1. Baseline comparison between MPP patients and healthy controls

The 2 groups were comparable in terms of age, sex, and body weight (all *P* > .05). However, the MPP cohort demonstrated significant elevations in white blood cell count, neutrophil count, and serum LDH levels (all *P* < .05; Table 1).

**Table 1:** Baseline characteristics of children in the MPP group and the healthy control group.

Indicator	MPP group (n = 364)	Control group (n = 160)	*Z*/χ^2^/*t*	*P*
Age (y)	7 (5, 8)	6 (5, 8)	−0.117*	.907
Sex, n (%)			0.005†	.942
Male	224 (61.54)	99 (61.88)		
Female	140 (38.46)	61 (38.12)		
Body weight (kg)	19.39 ± 1.49	19.48 ± 1.49	−0.696‡	.487
White blood cell count (×10^9^/L)	7.47 ± 1.97	6.14 ± 1.37	8.902‡	.000
Neutrophil count (%)	58.05 ± 9.72	44.95 ± 8.73	15.275‡	.000
LDH (ng/mL)	371.28 ± 47.13	316.95 ± 41.32	13.266‡	.000

LDH = lactate dehydrogenase, MPP = *Mycoplasma pneumoniae* pneumonia.

* Denotes *Z* value.

† Denotes χ^2^ value.

‡ Denotes *t* value.

### 3.2. Comparison of clinical characteristics between MPP children with good and poor prognosis

Baseline demographics and routine laboratory parameters, including age, sex, and perinatal history, were comparable between the good- and poor-prognosis groups (all *P* > .05). In contrast, children with a poor outcome exhibited significantly elevated levels of inflammatory and disease-severity markers, such as fever duration/peak, CPIS, MP antibody titer, LDH, D-D, CEACAM6, and CEACAM8 (all *P* < .05; Table 2).

**Table 2:** Clinical characteristics of MPP patients stratified by outcome.

Variable	Good prognosis (n = 295)	Poor prognosis (n = 69)	*Z*/χ^2^/*t*	*P*
Age (y)	6 (5, 8)	7 (5, 8)	−0.472*	.637
Sex, n (%)			0.458†	.499
Male	184 (62.37)	40 (57.97)		
Female	111 (37.63)	29 (42.03)		
Birth history	19.40 ± 1.47	19.33 ± 1.55	−0.352‡	.725
Term			0.093†	.760
Preterm	239 (81.02)	57 (82.61)		
Duration of fever (d)	56 (18.98)	12 (17.39)		
Peak temperature (°C)	6 (4, 7)	7 (6, 8)	−4.867*	.000
≥38.5			11.236†	.001
<38.5	207 (70.17)	62 (89.86)		
CPIS (points)	88 (29.83)	7 (10.14)		
MP antibody titer	5 (5, 6)	9 (7, 10)	−9.533*	.000
1:80			17.305†	.000
≥1:160	237 (80.34)	39 (56.52)		
Platelet count (×10^9^/L)	58 (19.66)	30 (43.48)		
White blood cell count (×10^9^/L)	241.02 ± 19.81	244.62 ± 14.33	1.423‡	.156
Neutrophil count (%)	7.43 ± 1.97	7.65 ± 1.99	0.818‡	.414
Red blood cell count (×10^12^/L)	57.72 ± 9.84	59.48 ± 9.12	1.355‡	.176
Hemoglobin (g/L)	4.27 ± 0.41	4.35 ± 0.35	1.502‡	.134
LDH (ng/mL)	121.46 ± 9.84	122.25 ± 10.31	0.595‡	.552
D-D (ng/mL)	358.66 ± 40.86	425.21 ± 31.56	14.844‡	.000
Length of stay (d)	13.18 (11.66, 15.24)	26.84 (22.95, 33.21)	−12.536*	.000
CEACAM6 (pg/mL)	6 (5, 7)	7 (5, 7)	−1.125*	.261
CEACAM8 (pg/mL)	67.02 (62.07, 70.98)	75.92 (69.71, 84.20)	−7.001*	.000

CEACAM = carcinoembryonic antigen-related cell adhesion molecule, CPIS = Clinical Pulmonary Infection Score, D-D = D-dimer, LDH = lactate dehydrogenase, MP = *Mycoplasma pneumoniae*, MPP = *Mycoplasma pneumoniae* pneumonia.

* Denotes *Z* value.

† Denotes χ^2^ value.

‡ Denotes *t* value.

### 3.3. Independent predictors of poor outcome in childhood MPP

Multivariable logistic regression was performed with the following variables as independent predictors: duration of fever (continuous), peak body temperature ≥ 38.5°C (binary), CPIS (continuous), MP antibody titer ≥ 1:160 (binary), LDH (continuous), D-D (continuous), and serum CEACAM6 and CEACAM8 levels (continuous). Poor clinical outcome was the dependent variable (poor = 1, good = 0). Multicollinearity was excluded because the variance inflation factor for each covariate was <2. After adjustment, higher CPIS, MP antibody titer ≥ 1:160, elevated LDH, D-D, CEACAM6, and CEACAM8 were independently associated with poor prognosis (all *P* < .05; Table 3).

**Table 3:** Multivariable logistic regression analysis of factors associated with poor prognosis in pediatric MPP.

Variable	β	SE	Walds	*P*	OR	95% CI	VIF
CPIS score	1.031	0.131	62.281	.000	2.804	2.171–3.622	1.408
MP antibody titer	0.432	0.105	16.994	.000	1.540	1.254–1.892	1.037
LDH	0.034	0.016	4.620	.032	1.035	1.003–1.068	1.338
D-D	1.256	0.385	10.665	.001	3.511	1.652–7.459	1.681
CEACAM6	0.244	0.096	6.514	.011	1.276	1.058–1.539	1.286
CEACAM8	0.122	0.018	44.372	.000	1.129	1.090–1.171	1.215

CEACAM = carcinoembryonic antigen-related cell adhesion molecule, CI = confidence interval, CPIS = Clinical Pulmonary Infection Score, D-D = D-dimer, LDH = lactate dehydrogenase, MP = *Mycoplasma pneumoniae*, MPP = *Mycoplasma pneumoniae* pneumonia, OR = odds ratio, SE = standard error, VIF = variance inflation factor.

### 3.4. Predictive performance of admission CEACAM6 and CEACAM8 for poor outcome

**Figure 2.**
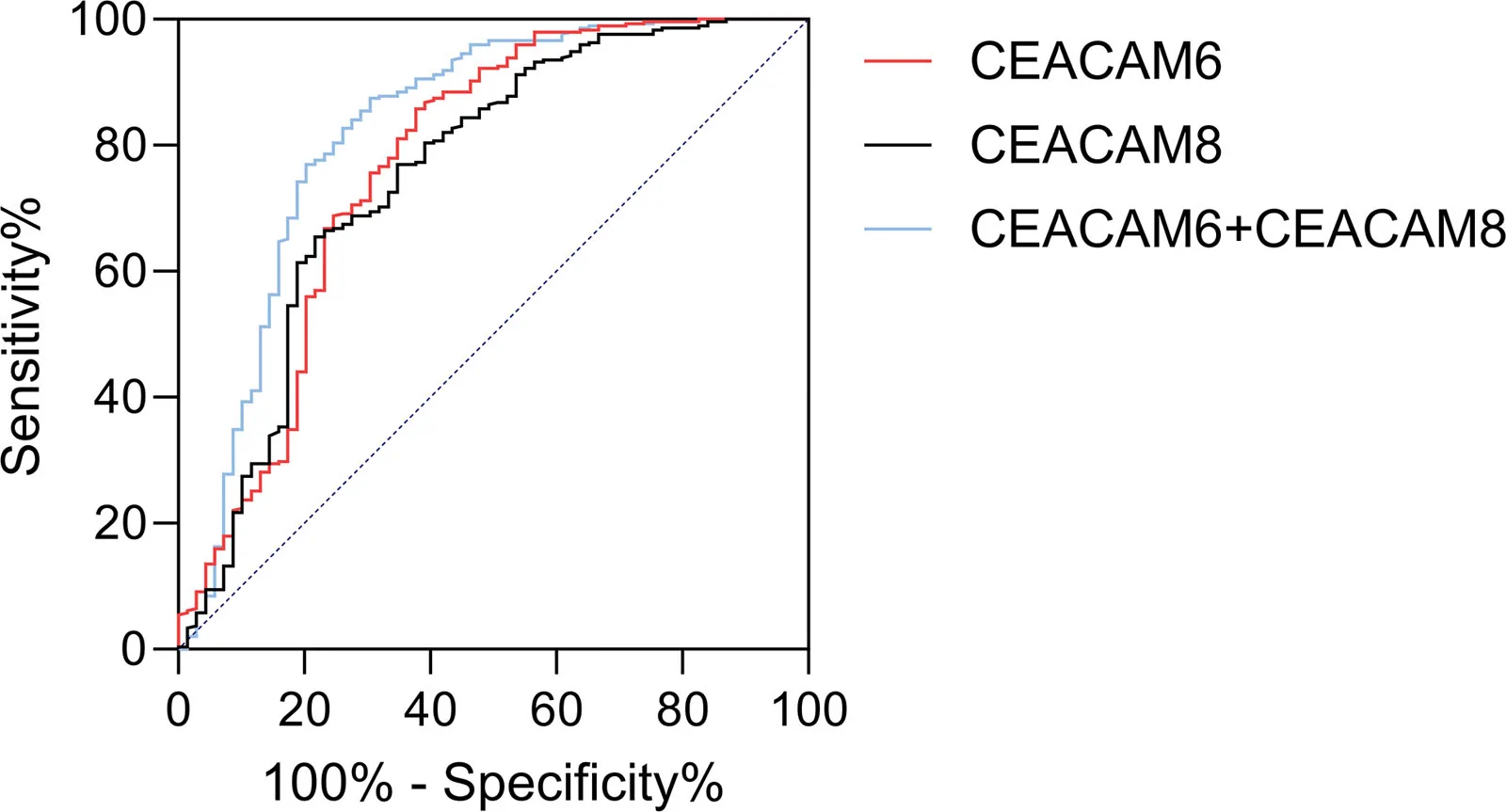
ROC curves evaluating the predictive value of admission CEACAM6 and CEACAM8 for poor prognosis in children with MPP. CEACAM = carcinoembryonic antigen-related cell adhesion molecule, MPP = *Mycoplasma pneumoniae* pneumonia, ROC = receiver-operating characteristic.

ROC analysis indicated that serum CEACAM6 levels at admission discriminated between good and poor prognosis (cutoff 73.37 ng/mL: sensitivity 62.3%, specificity 85.8%, area under the curve [AUC] 0.771 [95% CI = 0.697–0.844]). CEACAM8 showed comparable discriminatory ability (cutoff 55.57 ng/mL: sensitivity 78.3%, specificity 65.4%, AUC 0.755 [95% CI = 0.683–0.827]). Notably, the combination model demonstrated improved discrimination, with increased specificity (92.2%) and enhanced AUC (0.826 [95% CI = 0.758–0.894]), outperforming either individual marker (Fig. 2).

### 3.5. Temporal changes in inflammatory mediators and serum CEACAM6/CEACAM8

**Figure 3.**
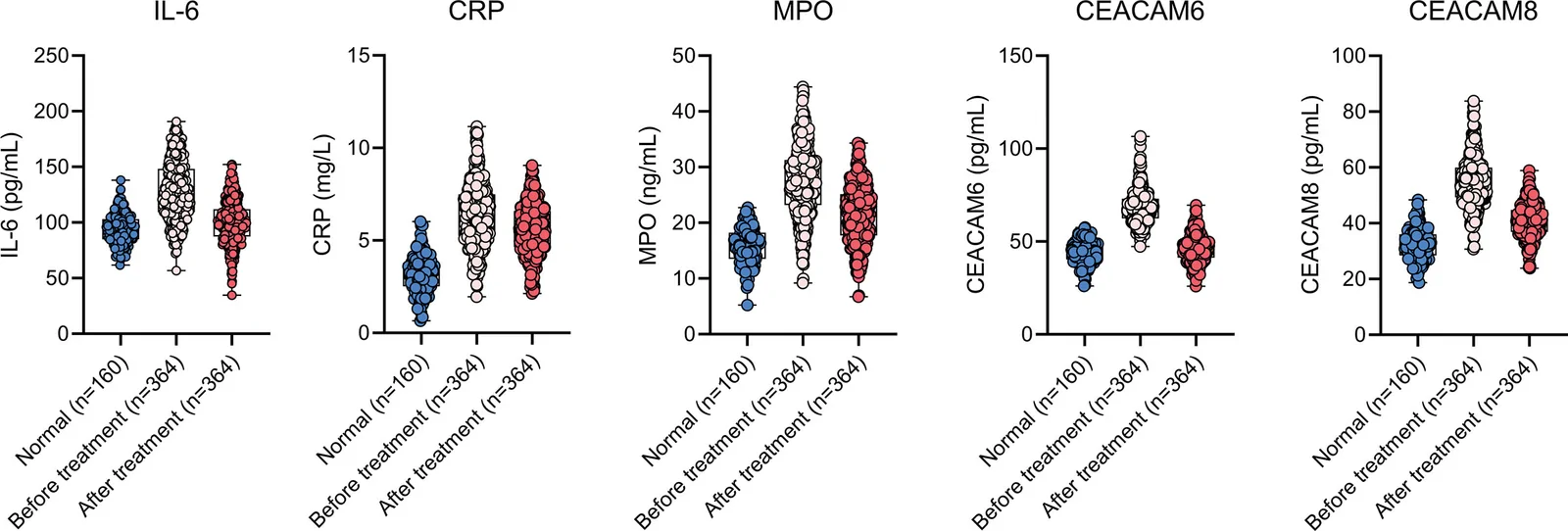
Temporal changes in inflammatory cytokines and serum CEACAM6/CEACAM8 levels before and after treatment. CEACAM = carcinoembryonic antigen-related cell adhesion molecule, CRP = C-reactive protein, IL-6 = interleukin-6, MPO = myeloperoxidase.

Serum levels of IL-6, CRP, MPO, CEACAM6 and CEACAM8 were markedly elevated in MPP patients versus controls and significantly decreased after antimicrobial therapy (all *P* < .05; Fig. 3).

### 3.6. Correlation of baseline CEACAM6 and CEACAM8 with inflammatory indices in MPP

**Figure 4.**
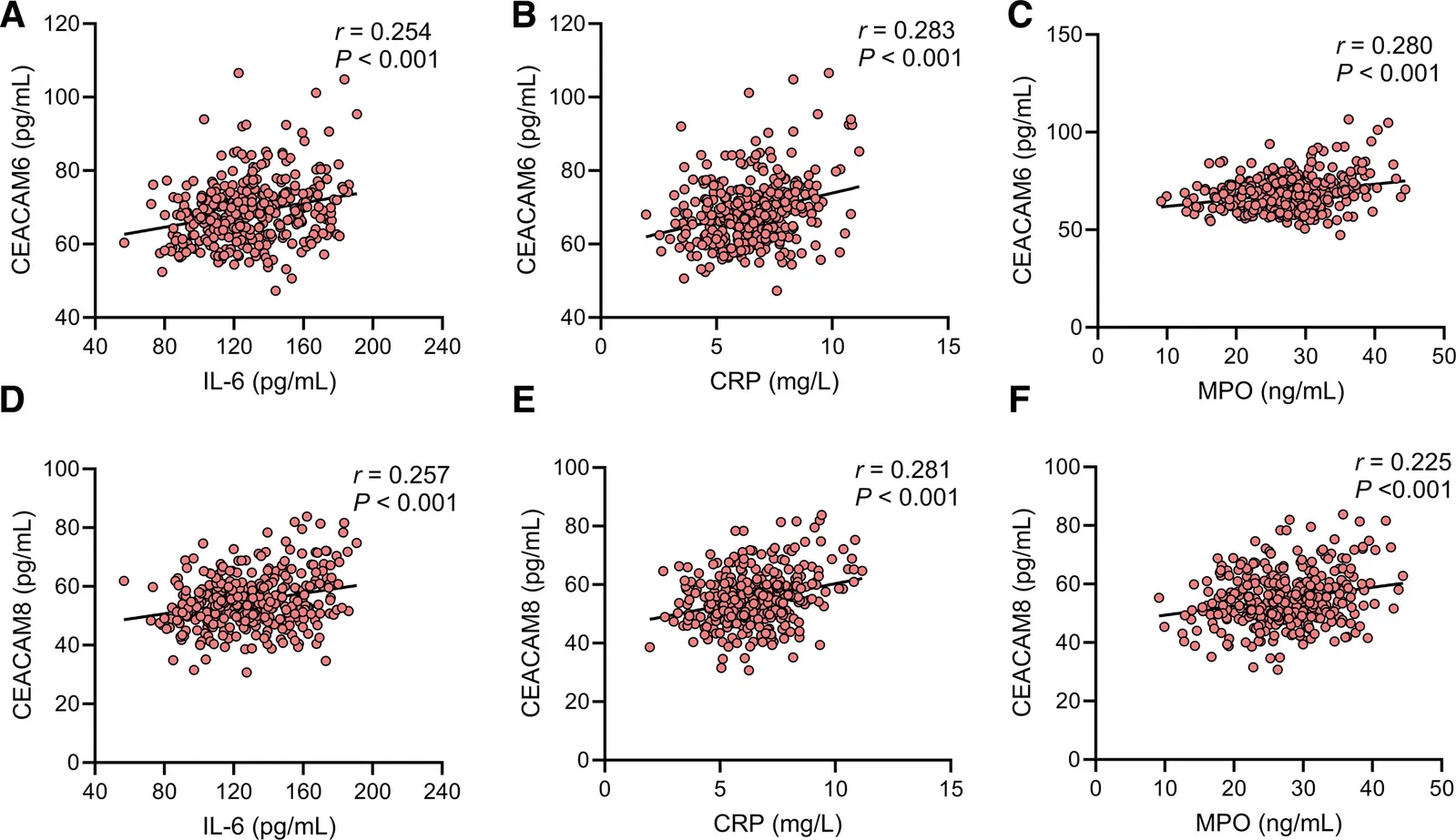
Correlation between pretreatment CEACAM6, CEACAM8, and inflammatory indices in MPP patients. CEACAM = carcinoembryonic antigen-related cell adhesion molecule, CRP = C-reactive protein, IL-6 = interleukin-6, MPO = myeloperoxidase, MPP = *Mycoplasma pneumoniae* pneumonia.

Pretreatment serum levels of both CEACAM6 and CEACAM8 demonstrated significant positive correlations with concentrations of IL-6, CRP, and MPO (all *P* < .05; Fig. 4).

## 4. Discussion

The present study identified serum CEACAM6 and CEACAM8 as potential biomarkers associated with poor prognosis in pediatric MPP, with their combination demonstrating favorable discriminatory ability. Compared with novel composite inflammatory indices recently proposed – including the lymphocyte-to-monocyte ratio, neutrophil-to-platelet ratio, and systemic immune-inflammation index – these conventional markers exhibit certain predictive efficacy yet lack underlying biological mechanistic substantiation. In contrast, the application of CEACAM6 and CEACAM8 for prognostic assessment in pediatric MPP in the present study not only demonstrates superior predictive performance but also validates their molecular mechanistic basis, thereby achieving dual value in both mechanistic elucidation and clinical prediction.^[17]^

In recent years, nucleic acid biomarkers have also garnered attention in MPP prognostic evaluation. Studies have demonstrated that the combination of long noncoding RNA NNT-AS1, CRP, and procalcitonin exhibits high accuracy (AUC 0.959) in predicting RMPP; however, nucleic acid marker detection imposes stringent sample processing requirements and exhibits limited clinical accessibility. As serum soluble proteins, CEACAM6 and CEACAM8 can be stably detected using standard ELISA methodologies, offering superior clinical translational feasibility.^[18]^ The marked heterogeneity in pediatric MPP outcomes reflects dysregulated neutrophil-mediated immune responses.^[19,20]^ Excessive neutrophil activation drives pulmonary injury through respiratory burst, degranulation, and neutrophil extracellular traps (NETs) formation,^[21]^ with elevated serum NETs recently shown to predict severe MPP with an AUC of 0.820^[22]^ – a performance comparable with our combined CEACAM6/CEACAM8 model.

CEACAM6 and CEACAM8 are glycosylphosphatidylinositol-anchored glycoproteins expressed on human neutrophils that interact with host CEACAM family members, bacterial and fungal pathogens, and cellular ligands to transmit and receive signals that modulate neutrophil function. CEACAM6 forms membrane complexes with CEACAM3, thereby amplifying CEACAM3-mediated activation and augmenting cytokine production.^[23]^ In other respiratory infections such as COVID-19, CEACAM6 and CEACAM8 have been implicated in dysregulated intercellular communication between developing neutrophils and type II alveolar epithelial cells, propagating inflammatory cascades and epithelial damage that accelerate disease progression. The CEACAM8–CEACAM6 regulatory network may exacerbate COVID-19 severity through promoting the differentiation of immature neutrophils, thereby contributing to the progression to acute respiratory distress syndrome.^[24]^

In the present study, serum CEACAM6 and CEACAM8 concentrations were markedly elevated in children with poor MPP outcomes relative to those with favorable outcomes, underscoring a close association between these biomarkers and prognosis. Multivariable logistic regression identified increased CEACAM6 and CEACAM8 levels as independent risk factors for poor outcome, while ROC analysis revealed high discriminatory accuracy, with AUCs of 0.771 and 0.755, respectively. Combining both biomarkers yielded an AUC of 0.826, significantly surpassing either alone. Collectively, these findings indicate that serum CEACAM6 and CEACAM8 represent robust prognostic indicators in pediatric MPP and that dual assessment offers superior predictive performance.

In MPP, cellular injury drives leukocytosis, neutrophilia, LDH elevation,^[25–27]^ and upregulated CRP^[28]^ and IL-6,^[29]^ all correlating with disease severity.^[30]^ Notably, MPO – a major NETs component – further propagates tissue injury via oxidative stress,^[31]^ collectively reflecting neutrophil-mediated pulmonary inflammation.^[25–27,31]^ In the present cohort, IL-6, CRP, MPO, CEACAM6, and CEACAM8 were all significantly higher than control values both before and after azithromycin therapy, but declined substantially following treatment, indicating that azithromycin effectively curtails MPP-related inflammation and improves outcome. Moreover, pretreatment CEACAM6 and CEACAM8 levels correlated positively with IL-6, CRP, and MPO, suggesting that these adhesion molecules modulate inflammatory networks that drive disease progression. Upregulation of CEACAM6 may, in particular, intensify pulmonary inflammation and thereby exacerbate lung injury.^[32]^

In summary, serum CEACAM6 and CEACAM8 concentrations are markedly elevated in children with MPP who experience poor outcomes and correlate positively with key inflammatory mediators, including IL-6, CRP, and MPO. The combined AUC of 0.826 for predicting adverse prognosis exceeds that of either biomarker alone, indicating that CEACAM6 and CEACAM8 represent promising prognostic biomarkers for pediatric MPP. Nevertheless, several limitations should be acknowledged. First, the single-center design and modest sample size may compromise statistical power and external validity. Second, serum CEACAM measurements were performed with a single analytical platform, introducing potential technical variability; moreover, the findings have not been replicated in an independent cohort, limiting robustness. Finally, although associations with inflammatory markers were identified, the underlying molecular mechanisms remain to be elucidated; future mechanistic studies are warranted to clarify the pathobiological roles of CEACAM6 and CEACAM8 in MPP.

## Author contributions

**Conceptualization:** Shenghua Ge, Lingyan Ma.

**Data curation:** Shenghua Ge, Dongqing Song.

**Investigation:** Shenghua Ge.

**Resources:** Shenghua Ge, Dongqing Song.

**Supervision:** Lingyan Ma.

**Formal analysis:** Lingyan Ma.

**Project administration:** Lingyan Ma.

**Methodology:** Shenghua Ge, Lingyan Ma.

**Validation:** Lingyan Ma, Dongqing Song.

**Writing – review & editing:** Lingyan Ma, Dongqing Song.

**Writing – original draft:** Shenghua Ge, Dongqing Song.

## References

[1] Zhou F Yang S Zhang W Huang M. Assessment of serum parameters caused by the outbreak of Mycoplasma pneumoniae pneumonia in children after COVID-19. Sci Rep. 2025;15:28306. 10.1038/s41598-025-13555-6 PMC12319097 40754552

[2] Zheng Y Li G Dai H Zhu Y. Macrolide-resistant Mycoplasma pneumoniae pneumonia in Chinese children: a retrospective study of clinical features and prognosis. Ital J Pediatr. 2025;51:289. 10.1186/s13052-025-02137-x PMC12542163 41126285

[3] Cheng J Liu Y Zhang G. Atelectasis predicts poor prognosis in pediatric macrolides-unresponsive Mycoplasma pneumoniae pneumonia with A2063/2064G mutations treated with azithromycin. Front Cell Infect Microbiol. 2025;15:1604102. 10.3389/fcimb.2025.1604102 PMC12245878 40654577

[4] Zhao Q Ji S Jiang H. Predictive value of plasma sB7-H3 and YKL-40 in pediatric refractory Mycoplasma pneumoniae pneumonia. Open Med (Wars). 2025;20:20241114. 10.1515/med-2024-1114 PMC11737367 39822987

[5] Gong C Yue H Li Q. Building a diagnostic prediction model for severe Mycoplasma pneumoniae pneumonia in children using machine learning. Front Public Health. 2025;13:1585042. 10.3389/fpubh.2025.1585042 PMC12488690 41048266

[6] Ding L Jiang Y. Biomarkers associated with the diagnosis and prognosis of Mycoplasma pneumoniae pneumonia in children: a review. Front Cell Infect Microbiol. 2025;15:1552144. 10.3389/fcimb.2025.1552144 PMC11958718 40171163

[7] Khairnar V Ngo VN. Editorial: role of carcinoembryonic antigen-related cell adhesion molecules in pathogen responses, tumorigenicity, and immune modulation. Front Immunol. 2024;15:1388453. 10.3389/fimmu.2024.1388453 PMC10949890 38504992

[8] Wu G Wang D Xiong F. The emerging roles of CEACAM6 in human cancer (Review). Int J Oncol. 2024;64:27. 10.3892/ijo.2024.5615 PMC10836497 38240103

[9] Zhao D Cai F Liu X. CEACAM6 expression and function in tumor biology: a comprehensive review. Discov Oncol. 2024;15:186. 10.1007/s12672-024-01053-6 PMC11127906 38796667

[10] Wu CY Cilic A Pak O. CEACAM6 as a novel therapeutic target to boost HO-1-mediated antioxidant defense in COPD. Am J Respir Crit Care Med. 2023;207:1576–90. 10.1164/rccm.202208-1603OC 37219322

[11] Singer BB Opp L Heinrich A. Soluble CEACAM8 interacts with CEACAM1 inhibiting TLR2-triggered immune responses. PLoS One. 2014;9:e94106. 10.1371/journal.pone.0094106 PMC3990526 24743304

[12] Sundarrajan S Sridhar KN Moorthy M Ramaswamy G. Study of immunological and inflammatory gene response in Indian cohort of COVID-19 patients by nanostring technology. Immunol Res. 2025;73:77. 10.1007/s12026-025-09626-5 40299133

[13] Ma L Ge S Song D. Predictive value of CCL26 and CCR3 levels for prognosis assessment in children with Mycoplasma pneumoniae pneumonia treated with azithromycin. Transl Pediatr. 2025;14:1806–15. 10.21037/tp-2025-296 PMC12433130 40949912

[14] Subspecialty Group of Respiratory, the Society of Pediatrics, Chinese Medical Association; China National Clinical Research Center of Respiratory Diseases; Editorial Board, Chinese Journal of Pediatrics. Evidence-based guideline for the diagnosis and treatment of Mycoplasma pneumoniae pneumonia in children (2023). Pediatr Investig. 2025;9:1–11. 10.1002/ped4.12469 PMC11998179 40241891

[15] Becerra-Hervás J Guitart C Covas A. The clinical pulmonary infection score combined with procalcitonin and lung ultrasound (CPIS-PLUS), a good tool for ventilator associated pneumonia early diagnosis in pediatrics. Children (Basel). 2024;11:592. 10.3390/children11050592 PMC11120099 38790587

[16] Zheng C Zheng Z Hu X Huang Y Han Y. Risk analysis of poor prognosis and follow-up observation of children with Mycoplasma pneumoniae pneumonia complicated by plastic bronchitis. Front Pediatr. 2025;13:1565773. 10.3389/fped.2025.1565773 PMC12245785 40656192

[17] Fan GZ Zhu YH Hu LX Qu ZH Liu YB. Predictive value of traditional and novel composite inflammatory indicators for severe and refractory Mycoplasma pneumoniae pneumonia in children. J Inflamm Res. 2025;18:16981–90. 10.2147/JIR.S561489 PMC12682924 41368352

[18] Chen P Huang Z Chen L. The relationships between LncRNA NNT-AS1, CRP, PCT and their interactions and the refractory Mycoplasma pneumoniae pneumonia in children. Sci Rep. 2021;11:2059. 10.1038/s41598-021-81853-w PMC7820231 33479472

[19] Yang Z Shi R Zhou X. Shifting epidemic trends and severity in pediatric Mycoplasma pneumoniae infections in the post-COVID-19 era. Ital J Pediatr. 2025;51:219. 10.1186/s13052-025-02064-x PMC12247333 40640857

[20] Rizo-Téllez SA Sekheri M Filep JG. Myeloperoxidase: regulation of neutrophil function and target for therapy. Antioxidants (Basel). 2022;11:2302. 10.3390/antiox11112302 PMC9687284 36421487

[21] Zhu Y Luo Y Li L. Immune response plays a role in Mycoplasma pneumoniae pneumonia. Front Immunol. 2023;14:1189647. 10.3389/fimmu.2023.1189647 PMC10250694 37304280

[22] Fan X Jiang J. Neutrophil extracellular traps as a predictive biomarker for severe Mycoplasma pneumonia in children: a clinical observational study. BMC Res Notes. 2025;18:394. 10.1186/s13104-025-07334-9 PMC12486646 41029340

[23] Skubitz KM. The role of CEACAMs in neutrophil function. Eur J Clin Invest. 2024;54:e14349. 10.1111/eci.14349 PMC11646290 39674879

[24] Huang R Meng T Zha Q. The predicting roles of carcinoembryonic antigen and its underlying mechanism in the progression of coronavirus disease 2019. Crit Care. 2021;25:234. 10.1186/s13054-021-03661-y PMC8254455 34217339

[25] Zhang Z Wan R Yuan Q. Cell damage and neutrophils promote the infection of Mycoplasma pneumoniae and inflammatory response. Microb Pathog. 2022;169:105647. 10.1016/j.micpath.2022.105647 35724831

[26] Huang X Qin H Zhang R Jia X Zhao D Liu F. Neutrophils are involved in the development and outcomes of plastic bronchitis associated with Mycoplasma pneumoniae pneumonia. Respir Res. 2025;26:92. 10.1186/s12931-025-03167-z PMC11890523 40057734

[27] Bertilacchi MS Vannucci G Piccarducci R. Serum lactate dehydrogenase levels reflect the lung injury extension in COVID-19 patients at hospital admission. Immun Inflamm Dis. 2025;13:e70168. 10.1002/iid3.70168 PMC11898011 40071734

[28] Li L Zhang Y Zhao L Shi Y. C-reactive protein-induced injury in Mycoplasma pneumoniae-infected lung epithelial cells is mediated by the P38 MAPK/mitochondrial apoptosis pathway. Microbiol Spectr. 2025;13:e0162624. 10.1128/spectrum.01626-24 PMC11878036 39932324

[29] Zhang Z Wang P Lei T. The role and impact of the IL-6 mediated JAK2-STAT1/3 signaling pathway in the pathogenesis of gout. Front Pharmacol. 2025;16:1480844. 10.3389/fphar.2025.1480844 PMC11959054 40170729

[30] Liu L Wenjie X Wang Y Wang X. Threshold effect of white blood cell count on the risk of refractory Mycoplasma pneumoniae pneumonia in pediatric patients: a retrospective cohort study. Medicine (Baltimore). 2025;104:e44999. 10.1097/MD.0000000000044999 PMC12499730 41054137

[31] Lin W Chen H Chen X Guo C. The roles of neutrophil-derived myeloperoxidase (MPO) in diseases: the new progress. Antioxidants (Basel). 2024;13:132. 10.3390/antiox13010132 PMC10812636 38275657

[32] Rinchai D Chaussabel D. Assessing the potential relevance of CEACAM6 as a blood transcriptional biomarker. F1000Res. 2022;11:1294. 10.12688/f1000research.126721.2 PMC11375406 39239252

